# The VTA dopaminergic system as diagnostic and therapeutical target for Alzheimer's disease

**DOI:** 10.3389/fpsyt.2022.1039725

**Published:** 2022-10-17

**Authors:** Paraskevi Krashia, Elena Spoleti, Marcello D'Amelio

**Affiliations:** ^1^Department of Experimental Neurosciences, IRCCS Santa Lucia Foundation, Rome, Italy; ^2^Department of Science and Technology for Humans and the Environment, Università Campus Bio-Medico di Roma, Rome, Italy; ^3^Department of Medicine and Surgery, Università Campus Bio-Medico di Roma, Rome, Italy

**Keywords:** neuropsychiatric symptoms, mesolimbic, mesocortical, ventral tegmental area, midbrain, preclinical AD, DAT Scan, Tg2576

## Abstract

Neuropsychiatric symptoms (NPS) occur in nearly all patients with Alzheimer's Disease (AD). Most frequently they appear since the mild cognitive impairment (MCI) stage preceding clinical AD, and have a prognostic importance. Unfortunately, these symptoms also worsen the daily functioning of patients, increase caregiver stress and accelerate the disease progression from MCI to AD. Apathy and depression are the most common of these NPS, and much attention has been given in recent years to understand the biological mechanisms related to their appearance in AD. Although for many decades these symptoms have been known to be related to abnormalities of the dopaminergic ventral tegmental area (VTA), a direct association between deficits in the VTA and NPS in AD has never been investigated. Fortunately, this scenario is changing since recent studies using preclinical models of AD, and clinical studies in MCI and AD patients demonstrated a number of functional, structural and metabolic alterations affecting the VTA dopaminergic neurons and their mesocorticolimbic targets. These findings appear early, since the MCI stage, and seem to correlate with the appearance of NPS. Here, we provide an overview of the recent evidence directly linking the dopaminergic VTA with NPS in AD and propose a setting in which the precocious identification of dopaminergic deficits can be a helpful biomarker for early diagnosis. In this scenario, treatments of patients with dopaminergic drugs might slow down the disease progression and delay the impairment of daily living activities.

## Introduction

Alzheimer's Disease (AD) is the prevailing form of age-associated dementia, accounting for approximately 60–80% of cases (1–3). The neuropathology of AD and the progressive cognitive decline are highly linked to the presence amyloid-beta (Aβ) and neurofibrillary tangles, whose deposition in the brain first appears in neocortical regions involved in cognition—mainly the entorhinal cortex and the hippocampal formation—and later spreads to neural hubs that underlie motor and sensory structures, planning, emotion and spatial navigation (4–10).

Neuropsychiatric symptoms (NPS) are also particularly common in the course of the disease, affecting up to half of patients with Mild Cognitive Impairment (MCI) and nearly all patients with AD-dementia (11–14). These symptoms mainly include depression, anxiety, psychosis, agitation, irritability and sleep disturbances, although apathy—described as loss of motivation, decreased interest and lack of emotional reactivity (15)—is the most common of these symptoms, with a prevalence in approximately 50% of MCI and 80% of AD patients (16–20).

Due to their appearance since the MCI stage, apathy and other NPS have an important prognostic value, as they help to predict the incidence of dementia, conversion from MCI to AD, and the acceleration in disease progression and cognitive decline (13, 21–25). However, the manifestation of these symptoms is not to be underestimated as they contribute significantly to worsening the daily functioning and disability of patients, they accelerate and prolong hospitalization and financial burden, and they worsen caregiver distress due to increased reliance of patients for everyday living (26, 27). Of note, in line with the faster disease progression in patients with NPS, recently an association was observed between apathy and an increased risk of mortality in dementia and elderly people (28, 29). Thus, interventions focused on treating NPS could have an important impact on patients, caregivers, and society. As such, an increased knowledge of the biological mechanisms underlying apathy and other symptoms in AD would result in a better perception of the disease and its etiology, and could lead to earlier and more efficient treatments and care planning.

The rising interest in NPS observed in MCI and early AD patients led to a number of investigations of brain structure in post-mortem tissue and in metabolic and neuroimaging studies, to correlate brain alterations with NPS, with main focus on apathy and depression. These studies appear to highlight abnormalities/deficits mainly in the medial frontal cortex, as well as in other brain regions such as the posterior and anterior cingulate cortex, medial orbitofrontal cortex and the ventral striatum for the development of apathetic or depressive behavior in subjects with MCI and AD (30–39). Of note, all of these regions are interconnected. More importantly still, each region receives monosynaptic dopaminergic projections from the ventral tegmental area (VTA) of the midbrain. The widespread involvement, in apathy and depression, of these brain regions modulated by dopamine perhaps makes it unsurprising that these NPS are considered, at least in part, hypodopaminergic symptoms (33, 40).

In this Perspective, we will examine in more detail the particular connections between the dopaminergic VTA and brain areas involved in the appearance of NPS and other relevant cognitive symptoms in AD. We will also describe the most recent preclinical and clinical studies that argue in favor of a precocious dysfunction of VTA dopaminergic neurons in AD and its contribution to the early appearance of NPS. Finally, we will propose a scenario in which the prompt identification of dopaminergic deficits since the MCI stage and an early dopaminergic-based pharmacological treatment of patients might be particularly beneficial for improvement of NPS, and therefore particularly relevant to test the effectiveness for slowing down the disease progression.

## The mesocorticolimbic dopamine system

The VTA (area A10 in humans) is comprised of a group of notoriously heterogeneous neurons located near the midline in the midbrain, in close proximity to the more lateral substantia nigra pars compacta (SNpc, A9). In addition to dopamine neurons, the VTA contains GABAergic and glutamatergic neurons, while some dopaminergic neurons were also shown to co-release dopamine together with glutamate or GABA (41, 42).

**Figure 1:**
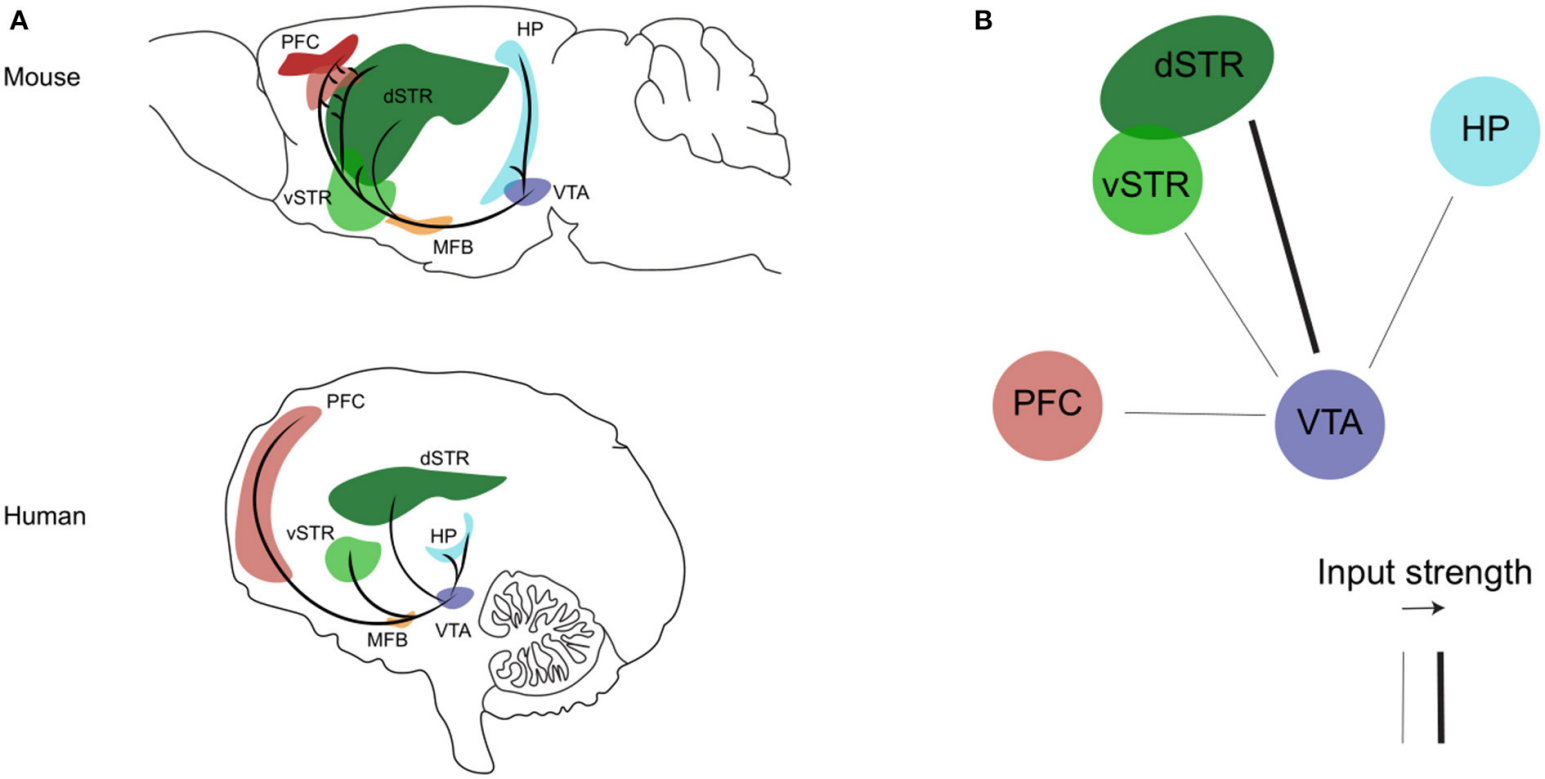
The mesocorticolimbic dopaminergic system. **(A)** Sagittal view of the mesocorticolimbic dopamine pathway and its principal target nuclei in the mouse (upper) and human (bottom) brain. **(B)** Representation of the impaired (thin line) dopaminergic inputs from the VTA described so far during the progression of AD (see text for details). Representations are adapted from the mouse and human brain atlas (Allen Institute). PFC, prefrontal cortex; HP, hippocampus; dSTR, dorsal striatum; vSTR, ventral striatum; MFB, medial forebrain bundle; VTA, ventral tegmental area.

The mesocorticolimbic dopaminergic pathway consists of cell bodies of dopamine neurons located in the VTA, that send ascending long-projection fibers rostrally toward limbic and cortical regions through the medial forebrain bundle, respectively forming the *mesolimbic* and *mesocortical* branches of the mesocorticolimbic system (Figure 1, Panel A). In recent years, the use of advanced tracing and optogenetics techniques in animals, and the improvement in imaging methods, have helped to well–define the circuit organization of the mesocorticolimbic system (41, 43–50). The most dense mesolimbic innervation from the dopaminergic VTA is at the level of the nucleus accumbens (NAc); these fibers then either remain in the NAc, innervating the core and shell subdomains, or they diverge to reach their terminal targets by extending toward the dorsal striatum and other limbic regions such as the amygdala and hippocampus. The mesocortical fibers instead, after going along the medial forebrain bundle, either bypass the NAc to extend dorsally toward the prefrontal cortex (PFC), or cross the NAc and dorsal striatum *en route* to the PFC. Of note, the mesocorticolimbic wiring is one of the last neuronal circuits of the brain to reach maturity. As observed both in rodents and non-human primates, this circuitry changes significantly not only during embryonic life and early postnatal development but also later on, to finally reach the mature form during adolescence (51–54). This peculiar feature of the mesocorticolimbic system is perhaps the basis for the increased vulnerability of this system to early-life stress and the many different positive or negative experiences that can drastically, and permanently, shape its development and function (55–58).

The vast connections of the mesocorticolimbic dopamine system with the various cortical, subcortical and limbic regions reflects the heterogeneity of its many functions, further enhanced by the functional and molecular heterogeneity of the VTA neurons themselves. In fact, the mesolimbic and mesocortical dopamine pathways have been shown to differ in their molecular markers, electrophysiological properties, anatomical organization and response to various stimuli (41, 59–63). This intrinsic variability and extensive neuronal connections of the mesocorticolimbic system allow for the vast range of its functions: indeed this system participates, amongst others, in brain functions like fear, aversion and reward, positive and negative reinforcement, motivated behavior, working-, place- and reward-associated memory, object recognition, decision-making, cognitive and executive functions, goal-directed behaviors, temporal control and food-intake (64–73). It is therefore not surprising that the mesocorticolimbic system has been highly implicated in a number of mood and neuropsychiatric disorders, ranging from anxiety, schizophrenia and depression to food and drug addiction, attention-deficit disorders and dementia (66, 72, 74–80).

## Dopamine in Alzheimer's disease

### Indirect evidence of mesocorticolimbic system dysfunctions in AD

It is well–known that the dopaminergic systems undergo several changes during normal aging. For example, decreased release of dopamine from its mesocorticolimbic terminals, reduced dopamine receptor expression, and in particular D2 receptors, or reduced dopamine transporter (DAT) expression in areas like the putamen, hippocampus and PFC are all features that are commonly observed in the human brain during physiological aging (80–83). Given the link between aging and dementia, it was not surprising that dopamine has intrigued neuroscientists also in relevance to AD (84–87). Thus, a number of earlier works provided evidence of reduced expression of dopamine receptors, DAT and tyrosine hydroxylase (TH; the rate-limiting enzyme necessary for dopamine synthesis) in post-mortem brains of people with AD, particularly in mesocorticolimbic areas such as the PFC, NAc and hippocampus (88–94). Additionally, these works demonstrated that at least 40% of AD cases also show pathological changes in the VTA and its dopamine neurons including Aβ plaques, tangles and decreased dopamine content (95–101). Although indirect, the evidence provided by these works support the notion that the mesocorticolimbic dopamine system undergoes changes in AD. Yet, such works could not answer the question of whether the deficits in the dopaminergic midbrain are an early event, or one of the many devastating consequences of brain damage occurring while the disease progresses.

### Early functional alterations of the mesocorticolimbic system in preclinical AD

Direct evidence supporting the contribution of dopaminergic system dysfunctions in early AD came from a preclinical study using the Tg2576 mouse model of AD. This mouse model overexpresses the human APPswe mutant (K670M/M671L) allele of familial AD (102). With more than 1,000 papers published over the last 20 years, this transgenic model is characterized by a particularly slow disease progression and has thus been essential for disentangling the early mechanisms underlying the disease (102–104). Indeed, Tg2576 mice develop early synaptic alterations in the hippocampus and cortex, reduced spine density of hippocampal pyramidal neurons, changes in hippocampal synaptic plasticity and deficits in memory and cognition that start since about 3 months of age (102–110).

The evidence linking the dopaminergic VTA with these early deficits in Tg2576 mice came after the observation of substantial apoptotic cell death of VTA dopamine neurons in these mice, accompanied by significant levels of local neuroinflammation, starting between 2–3 months of age and progressively worsening with age (111). The work by Nobili, et al., and subsequent papers, showed that the progressive degeneration of VTA TH^+^ neurons results in lower dopamine outflow in the hippocampus and correlates temporally with impairments in hippocampal neuronal function, synaptic plasticity, spatial memory and fear-associated memory performance (111–113). On the other hand, other brain regions containing TH^+^ neurons, in particular the SNpc and locus coeruleus (LC), are intact in Tg2576 mice at least until 6 months of age, suggesting that the early degeneration is selective for mesocorticolimbic neurons in the VTA and can account for the AD-like memory symptoms in these mice (114). Importantly, subchronic *in-vivo* treatment of Tg2576 mice with levodopa (L-DOPA; the natural dopamine precursor) or selegiline (an inhibitor of dopamine degradation by monoamine oxidase B) can completely rescue synaptic plasticity, pyramidal neuron excitability and memory deficits (111, 112), in line with previous evidence using different dopaminergic treatments (115–121). Similarly, a pro-autophagic treatment targeting the VTA dopamine neurons in Tg2576 mice, effective in delaying the neurodegenerative process, could also ameliorate the AD phenotype (122).

Since the early work in Tg2576 mice, alterations in the VTA, reductions of DAT in the hippocampus and loss of TH^+^ neurons were also observed in other mouse models of AD (3×Tg-AD, APPswe/PS11E9 and 5xFAD) (123–126). Overall, these works highlighted the dopaminergic VTA as a brain region associated with early AD, showing that the reduction of the mesocorticolimbic dopamine input precedes degeneration, inflammation or plaque formation in target areas, indicating also that hippocampal-related memory dysfunction in AD is secondary to VTA dopamine neuron degeneration. Of note, given the fact that the Tg2576 mouse overexpresses the APPswe mutation, one can assume that the early degeneration of the dopaminergic VTA can be associated with the higher levels of soluble and neurotoxic Aβ oligomers in this mouse, at a stage when insoluble Aβ (Aβ plaque deposition) is still undetectable (111). Indeed, Aβ oligomers have been found to impair autophagy and mitochondrial dynamics in AD, as demonstrated in cellular models and in dopaminergic neurons of the Tg2576 mouse model, thus contributing to cell death (122, 127, 128).

Importantly, the paper by Nobili et al., could explain not only memory deficits but also the occurrence of NPS in AD (111–114). This conclusion mainly derived from the evidence that the degeneration of VTA dopamine neurons in Tg2576 mice, and the sebsequent reduction of dopaminergic innervation in limbic brain areas related to reward, stress and emotional processing (129)—such as the NAc and ventral hippocampus—were associated with deficits during consumption of palatable food, affecting not only the ability of Tg2576 animals to favor an agreeable food such as chocolate over normal chow, but also reducing the overall levels of consumption. This depressive-like behavior in Tg2576 mice was completely rescued by selegiline, as sub-chronic treatment levels could improve impairments in mesolimbic reward processing by enhancing, in treated Tg2576 mice, the preference for chocolate eating and the amount of consumption (111). Although interpretations of results from animal behavioral studies, particularly in relevance to neuropsychiatric features of human diseases, are inherently complicated, this study could prove a first link between mesocorticolimbic dopamine deficits, neuropsychiatric-like deficits and AD at an early pre-plaque disease stage.

### Mesocorticolimbic system dysfunctions in MCI and AD

Given the small size of brainstem nuclei like the VTA (approximately 60,000 dopamine neurons in the adult human brain), the resolution power of imaging techniques in humans has been a limiting factor that likely contributed to the neglection of such small brain regions from AD screening in the past. The first study on human subjects showing direct evidence of a functional disconnection of the VTA with brain areas normally affected in AD was the study by Serra, et al. (130). The authors recruited large numbers of amnestic MCI and sporadic AD patients as well as healthy controls and used resting-state functional MRI (rs-fMRI at 3T) to assess the functional connectivity between the VTA and different brain areas (130). The study showed a significant disconnection between the VTA and the thalamus, medial-temporal regions and the parietal lobe in MCI patients. As the disease advanced to the AD stage, patients demonstrated additionally functional disconnection of the posterior parietal cortex with the LC. The disconnection from the VTA was mainly present in the hippocampus and parietal regions in MCI patients, while in AD it involved most regions of the default-mode network (130–132). A parallel study used structural MRI to link the volume of the VTA with typical AD clinical markers, in particular hippocampal size and memory index (133, 134). The main finding of the study was that the VTA volume is strongly associated with the size of the hippocampal formation and memory abilities of both AD patients and healthy controls, thus indicating that a smaller VTA size or shrinkage of the nucleus might correspond to a worse memory performance and smaller hippocampus. This conclusion did not involve the entire midbrain, since no association was observed between the SNpc and the size of the hippocampus in AD patients (133).

Interestingly, the study by Serra, et al. (130) was the first to observe a direct association between functional disconnections of the VTA and early NPS since the MCI stage (Table 1): comparison of patients with and without these symptoms confirmed that patients with apathy, depression or anxiety showed a stronger disconnection between the VTA and the default-mode network. Subsequent studies showed additional deficits that mostly affect the mesocorticolimbic system, while leaving the nigrostriatal system largely intact. For example, by combining structural MRI and ^18^F-FDG-PET glucose metabolic data, Iaccarino et al., showed significant tissue atrophy, reduced metabolism and widespread loss of gray matter in VTA targets in the medial temporal lobe related to memory performance, but also in targets related to NPS such as the ventral striatum, orbito-frontal cortices and amygdala in both MCI and AD patients (136). On the other hand, regions of the nigrostriatal pathway exhibited fewer alterations (136, 139). Similar results were obtained with a DAT-Scan [(123I)FP-CIT-SPECT], focusing specifically on the dopaminergic innervations (140).

**Table 1:** Summary of clinical studies investigating direct evidence for an association between deficits in the mesocorticolimbic dopamine system and NPS.

**Group subjects** **(Reference)**	**Screening** **method**	**Mesocorticolimbic** **alterations**	**Behavioral** **assessment** **method**	**NPS assessment results**
- 84 probable AD - 48 amnestic MCI (a-MCI) - 37 HS (130)	- resting-state fMRI at 3T	- a-MCI: lower FC in VTA and the right parietal lobule - AD: lower FC between VTA and posterior cingulate cortex, precuneus, parietal lobule.	NPI-12 (135).	- Apathy, depression, and anxiety were the most frequent NPS, no significant differences between AD and a-MCI. - Strong link between VTA connectivity and NPS (agitation, irritability, disinhibition) and sleep and eating disorders
- 60 AD-D - 53 MCI - 54 HS (136)	- structural MRI at 3T 18F-FDG-PET	- 77.7% of structures showing gray matter volume reductions in MCI and AD-D belong to the mesocorticolimbic DA pathway. - 58.3% of VTA targets present significant gray matter reductions, with most detectable at the MCI stage. - Regions of the Mesocorticolimbic pathway show significant metabolic connectivity changes in AD.	NPI-12	- Higher NPI total and depression scores vs. HS MCI: higher scores for anxiety, ADD higher scores in Apathy/ Indifference, aberrant motor behavior, appetite changes, Irritability. -Higher NPI scores associated with severe atrophy. Depression score correlated with atrophy in medial orbitofrontal cortex, anxiety with atrophy in the v. striatum, apathy with atrophy in HP, entorhinal area, para-HP gyrus and amygdala.
- 29 MCI, 34 AD-D with delusions - 29 MCI, 34 AD-D with no delusions - 63 HS (137)	- structural MRI at 1.5 or 3T	- greater gray matter loss in left and right caudate nuclei in patients who later developed delusions.	- NPI-12 - NPI-Questionare [NPI-Q; (138)].	- subjects with delusion presented with higher NPI scores already 1 year before the manifestation of symptoms. - greater longitudinal gray matter loss in delusional patients was observed in the bilateral medio-temporal areas (bilateral para-HP gyri and left HP) and in the right anterior cingulate cortex belonging to the mesocorticolimbic dopaminergic pathway.

AD-D, Alzheimer's with Dementia; HS, Healthy subjects; FC, functional connectivity, NPI-12, Neuropsychiatric Inventory; HP, hippocampus.

An interesting finding was that the degree of loss of dopaminergic inputs in the various brain targets examined was analogous between the MCI and AD-with-dementia stages, suggesting that the dopaminergic dysfunction is an early event that also reaches a plateau early along the disease progression (140). Of note, these studies also confirmed that the extend of gray matter atrophy in mesocorticolimbic dopaminergic targets since the MCI stage was associated with greater severity of NPS, in particular depression linked to the meso-cortical route, anxiety linked to the mesolimbic route, and apathy linked to the mesohippocampal/meso-amygdaloid route (136). Indeed, stronger loss of gray matter in dopaminergic pathways was also paralleled by a worsening not only of episodic memory but also of behavioral alterations, related to the emergence of psychiatric symptoms and delusions in patients with AD (137). Given the link between the mesocorticolimbic dopamine and NPS, and the fact that these symptoms speed up the disease progression, it is not surprising that functional disconnection of the VTA with mesocorticolimbic targets can accelerate the conversion from the MCI stage to clinical AD (137, 141, 142).

## Conclusions: Implications for early diagnosis and treatment

Overall, the above mentioned clinical studies show that the various functional, structural or metabolic alterations affecting the dopaminergic VTA and its mesocorticolimbic targets since the MCI stage (Figure 1, panel B) are a very precocious phenomenon in the disease progression, that can be related with the early appearance of NPS. In fact, today the assessment of CSF biomarkers and the application of the Neuropsychiatric Inventory (NPI) for evaluation of NPS are standard clinical practices, with a strong proven link between the two, such as increased levels of total (t-tau) and phosphorylated tau (p-tau) proteins and low levels of Aβ42 correlating with apathy, anxiety, agitation, and irritability (143–148). Combined with these diagnostic tools, early identification of alterations in the mesocorticolimbic dopamine system could provide an additional valid biomarker aimed at the prognosis of NPS and thus prediction of disease acceleration and faster conversion from MCI to AD.

Another aspect to consider is the possibility of early intervention based on a pharmacological treatment against dopamine-related symptoms since the MCI stage, with the aim of slowing down their appearance, improving the quality of life and delaying the conversion to AD. Several dopaminergic drugs have already shown to have positive effects in mild-AD patients (80, 149–153), but their effectiveness as agents that can delay conversion from MCI to AD has yet to be investigated. Also, these earlier clinical trials with dopaminergic drugs have focused mainly on cognitive rather that neuropsychiatric deficits. Yet, today there is increasing demand for the approval of specific drugs for the treatment of NPS in AD, considering also that no such drugs have been approved so far by the FDA. Indeed, drugs usually prescribed to patients with NPS are often anxiolytics, atypical antipsychotics, antidepressants or mood stabilizers, all unapproved for AD, with uncertain efficacy and important adverse effects (154–158). Thus, focused clinical trials that are specific for NPS in MCI and AD are essential. Fortunately, progress in this field is starting to emerge with specific focus on catecholamine reuptake inhibitors like methylphenidate (159–162).

Since disease-modifying treatments have failed, new studies need to focus on a paradigm shift for preventing and treating AD. The emerging experimental and clinical results pinpoint the VTA atrophy as a supportive feature for the diagnosis of probable AD and a target for next pharmacological treatment.

## Data availability statement

The original contributions presented in the study are included in the article/supplementary material, further inquiries can be directed to the corresponding author/s.

## Author contributions

All authors listed have made a substantial, direct, and intellectual contribution to the work and approved it for publication.

## Funding

PK was supported by an under-40 grant from the Italian Association for Alzheimer's Research [AIRALTZH-AGYR2020], by the Italian Ministry of Health [Research Grant: GR-2019-12370446] and by the American Alzheimer's Association [AARG-22-922961]. ES was supported by a PhD Fellowship by Fondazione Melchiorri (IT) and by the Strategic University Projects—Young Researcher Independence Grant [YRG2021] from the University Campus Bio-Medico (Rome, Italy). MD'A was supported by the American Alzheimer's Association [AARG-18-566270; AARG-21-851219], by the Italian Ministry of Health [Research Grant: RF-2018-12365527] and by Fondazione Roma (Rome, Italy).

## Conflict of interest

The authors declare that the research was conducted in the absence of any commercial or financial relationships that could be construed as a potential conflict of interest.

## Publisher's note

All claims expressed in this article are solely those of the authors and do not necessarily represent those of their affiliated organizations, or those of the publisher, the editors and the reviewers. Any product that may be evaluated in this article, or claim that may be made by its manufacturer, is not guaranteed or endorsed by the publisher.
